# Effects of physical exercise associated with a diet enriched with natural antioxidants on cerebral hypoperfusion and reperfusion injury in spontaneously hypertensive rats

**DOI:** 10.3389/fphys.2023.1091889

**Published:** 2023-01-23

**Authors:** Dominga Lapi, Giuseppe Federighi, Maria Serena Lonardo, Martina Chiurazzi, Espedita Muscariello, Giancarlo Tenore, Antonio Colantuoni, Ettore Novellino, Rossana Scuri

**Affiliations:** ^1^ Department of Biology, University of Pisa, Pisa, Italy; ^2^ Department of Translational Research on New Technologies in Medicine and Surgery, University of Pisa, Pisa, Italy; ^3^ Department of Clinical Medicine and Surgery, University of Naples “Federico II”, Naples, Italy; ^4^ Department of Pharmacy, University of Naples “Federico II”, Naples, Italy; ^5^ Hospital “A. Gemelli”, Catholic University, Rome, Italy

**Keywords:** hypertension, hypoperfusion, physical exercise, pial microcirculation, polyphenols, reperfusion, ROS

## Abstract

Oxidative stress is implicated in the pathogenesis of arterial hypertension. The reduction in the bioavailability of nitric oxide (NO) causes endothelial dysfunction, altering the functions of cerebral blood vessels. Physical exercise and intake of antioxidants improve the redox state, increasing the vascular NO production and/or the decrease in NO scavenging by reactive oxygen species (ROS). The present study was aimed at assessing the effects of physical exercise associated with a diet enriched with antioxidants from the Annurca apple in preventing the microvascular damage due to cerebral hypoperfusion and reperfusion injury in spontaneously hypertensive rats (SHRs). The rat pial microcirculation was investigated by intravital fluorescence microscopy through a parietal closed cranial window. As expected, SHRs subjected to physical exercise or an antioxidants-enriched diet showed a reduction of microvascular permeability, ROS formation, and leukocyte adhesion to venular walls, with a major effect of the antioxidants-enriched diet, when compared to untreated SHRs. Moreover, capillary perfusion was preserved by both treatments in comparison with untreated SHRs. Unexpectedly, the combined treatments did not induce higher effects than the single treatment. In conclusion, our results support the efficacy of physical activity or antioxidant supplement in reducing the microvascular alterations due to hypertension and ascribe to an antioxidants-enriched diet effective microvascular protection in SHRs.

## Introduction

Large amounts of data indicate that the increased arterial blood pressure on the vessel walls is correlated to a greater risk of cardiovascular damage, such as brain ischemic injury (Iadecola and Davisson, 2008; Pires et al., 2013; Webb and Werring 2018). It has been reported that about 87% of transient ischemic attacks/minor strokes (TIAs/MSs) are caused by narrowed or obstructed blood vessels in the brain cutting off the blood flow to neurons (Ganzer et al., 2016; Yu and Coutts 2018; Panuganti et al., 2022). Several studies demonstrate that the pathogenesis of hypertension is associated with oxidative stress, with a reduction in the bioavailability of nitric oxide (NO) accompanied by endothelial dysfunction and alterations in the structure of cerebral blood vessels, disrupting complex vasoregulatory mechanisms (Atochin and Huang 2011; Zhu et al., 2016; Quick et al., 2021; Ambrosino et al., 2022).

In any biological system, there is a balance between reactive oxygen species (ROS) or reactive nitrogen species (RNS) formation and their removal (Di Meo et al., 2016; Lushchak and Lushchak 2021). Gavazzi and coworkers showed that genetic deficiencies in ROS-generating enzymes in mice are accompanied by lower blood pressure compared with their wild-type counterparts (Gavazzi et al., 2007). The imbalance between elevated ROS (e.g., superoxide, hydrogen peroxide, and hydroxyl radical) production and/or reduced antioxidant capacity at the systemic level is accompanied by localized changes in regional circulations (Taniyama and Griendling 2003; Touyz and Schiffrin 2004; Alhayaza et al., 2020). In particular, it has been reported that ROS modulate numerous pathways involved in the control of systemic vascular resistance and blood pressure (Virdis et al., 2011; Oparil et al., 2018; Shi et al., 2022). Nevertheless, in any condition, the oxidative stress appears to play an important role in hypertension (Gonzàlez et al., 2014; Förstermann et al., 2017). Consequently, there is increasing evidence that oxidative stress is one of the targets for therapeutic interventions in the treatment of hypertension.

Several reports indicate that in hypertensive subjects, physical exercise improves the redox state by increasing the vascular NO production and/or decrease in NO scavenging by ROS, leading to endothelial adaptations (Gallanagh et al., 2011; Tofas et al., 2020; Pescatello et al., 2021). These adaptations are also reported in vascular beds of tissues that are inactive or less active during exercise; the involvement of humoral factors has been suggested (e.g., insulin), while active muscles release cytokines and peptides, termed myokines, exerting anti-inflammatory effects (Padilla et al., 2011; Codella et al., 2015).

Furthermore, epidemiologic studies have shown that a high intake of antioxidants, such as polyphenols and vitamins with higher serum concentrations, reduces the risk of cardiovascular diseases (Tressera-Rimbau et al., 2017; Cory et al., 2018). The bioactivity of flavonoids includes different mechanisms, such as vasodilation stimulation. The flavonoids, indeed, have been demonstrated to affect key cardiometabolic risk variables, such as lipid profile, blood glucose, blood pressure, and metabolic syndrome. Our previous results indicate that the administration of antioxidant polyphenols of natural origin can protect cerebral microcirculation from damage induced by ischemia and reperfusion injury (Lapi et al., 2015; Mastantuono et al., 2015; Lapi et al., 2016; Mastantuono et al., 2016; Lapi et al., 2020).

Previously, we observed that physical activity and a diet enriched with antioxidants individually have an important hypotensive effect, and the association showed a more prolonged, but not better, hypotensive effect than the single treatment (Del Seppia et al., 2022). In the present descriptive study, we assessed the effects of a physical exercise program associated with a diet enriched with products based on Annurca apple polyphenolic fraction (Maisto et al., 2022) on preventing microvascular damages due to cerebral hypoperfusion and reperfusion injury in spontaneously hypertensive rats (SHRs) in association or when administered alone.

We used the SHRs, as an experimental model, because the animals show interesting characteristics: they are normotensive at birth and progressively develop elevated blood pressure levels from the fifth week of life. We induced a transitory bilateral common carotid artery occlusion (BCCAO), and we evaluated the main indices of vascular damage, such as microvascular permeability increase, ROS formation, leukocyte adhesion, and perfused capillary length. These parameters were evaluated under baseline conditions and after hypoperfusion and reperfusion. Moreover, we evaluated the response to BCCAO in young SHRs (still in normotensive conditions) and in SHRs aged for a time corresponding to the duration of the treatments.

## Materials and methods

### Experimental design

Spontaneously hypertensive rats (SHRs) (Charles River, Calco, Italy) were used, which were about 4 weeks old when they arrived at our housing. All the animals were kept in a controlled environment at a constant temperature (24°C ± 1°C) and humidity (60 ± 5%), subjected to an artificial circadian cycle of 12 h day/night with free access to food and water.

The rats were treated according to the rules dictated by the Guide for the safety and use of laboratory pets (NIH publication 68-23 revised in 1985) and by the local university ethics committee and by the Ministry of Health (authorization no. 156/2017-PR).

The animals were randomly assigned to the following groups: 1) rats subjected to a moderate exercise program for 6 weeks (E group, *n* = 11); 2) rats subjected to a diet enriched with antioxidants for 6 weeks (AED group, *n* = 11), and 3) rats subjected to a moderate exercise program for 6 weeks with an associated diet enriched with antioxidants (E + AED group, *n* = 11). Twenty-two SHRs did not receive any treatment: 11 were used after 1 week of housing (at 5 weeks of age, Y group) to evaluate microvascular damage induced by hypoperfusion and reperfusion injury under normotensive conditions, while the remaining 11 were subjected to hypoperfusion and reperfusion when they were 12 weeks old (A group), corresponding to the age of the animals submitted to a treatment. Six animals belonging to each experimental group were used to determine the *in vivo* formation of reactive oxygen species (ROS) by 2’-7’-dichloro-fluorescein-diacetate (DCFH-DA) assay after hypoperfusion (*n* = 3) or after reperfusion (*n* = 3). Five animals of each group were monitored for microvascular damage.

## Exercise protocol

Trained animals performed moderate exercise for 6 weeks (Neto et al., 2015), three times a week for a total of 18 sessions, starting from the sixth week of life. In each session, the rats were taken from the housing room to the training room; after 15 min of acclimatization, the animals were manually placed inside a specially constructed training device consisting of a plastic wheel in which a corridor was delimited; and the rats were forced to run, following the rotational movement of the wheel, as previously described by Franzoni et al. (2017). The animals were trained to use the device for one week: for the first two days, they were allowed to explore and move around inside the wheel; successively, the wheel was driven slowly (4–5 m/min) to allow the rats to coordinate their pace with the apparatus’s rotation speed. After 3–4 days of adaptation, the rats were able to follow the movement of the wheel, running continuously for 30 min: the animals were ready to perform the exercising protocol.

The researcher planned the movement of the wheel, choosing the direction of rotation (clockwise or counterclockwise), the rotation speed (expressed in m/min), and the rotation time (min). Appropriate software programmed on the LAB VIEW system (National Instruments SRL, Milan, Italy) allowed us to ascertain the space traveled (m) in real time. The data were stored on an Excel table for the subsequent off-line analysis.

The rats exercised between 9:00 a.m. and 3:00 p.m. to reduce circadian influences.

**FIGURE 1:**
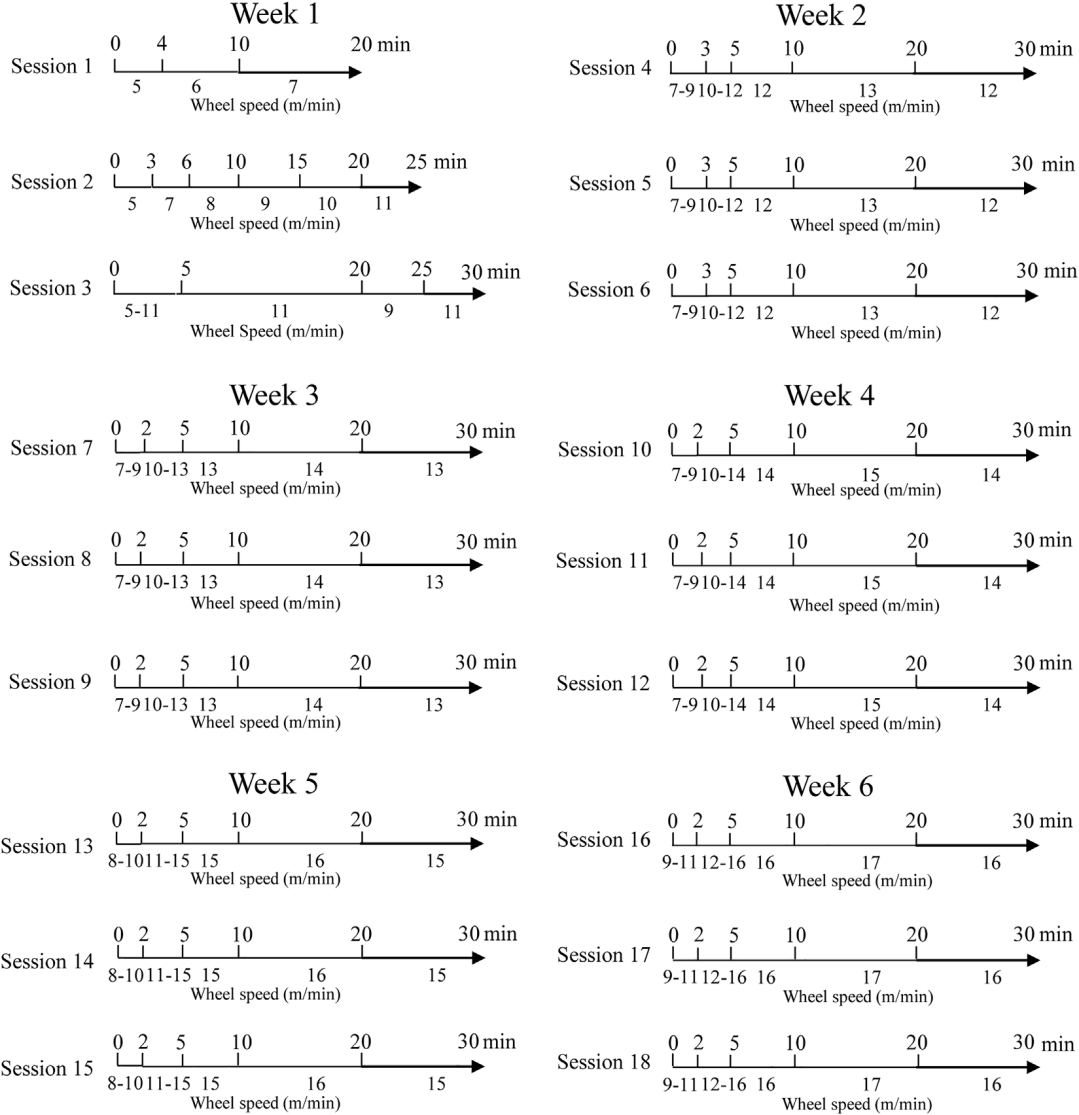
Exercising session planning.

The program was scheduled as shown in Figure 1. In Table 1, we report the distance traveled by rats at the end of every exercising session (E and E + AED groups).

**TABLE 1:** Distance travelled by rats at the end of every exercising session.

Training session	Treatment					
	Exercise			Exercise + Antioxidants-enriched diet		
	Meter traveled		Rats (N)	Meter traveled		Rats (N)
	Mean	S.E.		Mean	S.E.	
1	101.4	9.33	11	106.8	9.34	11
2	192.3	21.69	11	186.4	20.15	11
3	267.2	27.06	11	242.2	22.79	11
4	285.6	32.46	11	281.0	26.79	11
5	311.1	20.94	11	307.5	20.24	11
6	315.1	19.33	11	306.9	20.24	11
7	338.6	20.21	11	337.5	19.90	11
8	339.7	20.55	11	340.4	20.66	11
9	340.5	20.75	11	337.7	19.84	11
10	366.6	19.62	11	360.6	21.25	11
11	360.9	19.60	11	348.6	28.16	11
12	369.2	18.08	11	350.9	30.31	11
13	379.4	23.45	11	366.3	35.05	11
14	377.2	31.73	11	391.1	18.19	11
15	375.8	34.46	11	391.7	18.52	11
16	394.7	38.96	11	414.7	18.42	11
17	427.3	17.47	11	369.9	19.71	11
18	435.9	18.69	11	386.6	15.89	11

## Polyphenol administration

The antioxidants-enriched diet consisted of the administration of a polyphenolic fraction, obtained through the application of a technological process (acid treatment), capable of freeing the insoluble bound polyphenols from Annurca apple food matrix and of increasing polyphenol bioaccessibility (Maisto et al., 2022). It was added to drinking water at a dosage of 30 mg/b.w. The chosen dosage was established through pilot experiments evaluating the minimum quantity, inducing an antioxidative effect as shown in Del Seppia et al. (2022).

## Surgical procedure

Animals were anesthetized by an intraperitoneal injection of α-chloralose (50 mg/kg b.w., i.p.) (Sigma-Aldrich, Milan, Italy) and maintained with supplemental injections (α-chloralose 30 mg/kg b.w., i.v.). They were tracheotomized and mechanically ventilated (Mastantuono et al., 2017). Subsequently, the right and left common carotid arteries were isolated to induce hypoperfusion by clamping (BCCAO). Two catheters were inserted into the left femoral artery and in the right femoral vein, respectively. The arterial catheter was used to draw blood samples every 30 min to assess blood gas concentration. The venous catheter was used to administer additional anesthesia and fluorescent tracers [fluorescein isothiocyanate bound to dextran, 70 kD MW, FITC dextran (FD 70, Sigma-Aldrich, Milan, Italy), 50 mg/100 g b.w., i.v. as 5% wt/vol solution in 3 min, administered just once at the beginning of an experiment after 30 min of the preparation stabilization; rhodamine 6G, 1 mg/100 g b.w. in .3 mL, was given as a bolus with supplemental injection throughout BCCAO and reperfusion (final volume, .3 mL/100 g^−1^h^−1^)].

To proceed with the preparation of the cranial window, rats were secured on a stereotaxic plate and heated to 37.0°C ± .5°C to maintain the normal body temperature (monitored by a rectal probe). Moreover, the values of respiratory CO_2_ and blood gases were recorded and maintained stably within physiological ranges. A closed cranial window prepared above the parietal cortex (Ngai et al., 1988) was used to observe the pial microcirculation after the removal of the dura mater as reported previously in detail (Mastantuono et al., 2017).

Hypoperfusion, lasting 30 min, was induced by the placement of two atraumatic microvascular clips on the previously isolated common carotid arteries. Afterward, the clamps were removed and the pial microcirculation was observed sequentially for 60 min (reperfusion).

### Fluorescence microscopy

The *in vivo* observation of the pial microcirculation was performed using a fluorescence microscope (Leitz Orthoplan) equipped with long-distance objectives [2.5x, numerical aperture (NA) .08; 10x, NA .20; 20x, NA .25; 32x, NA .40, a 10x eyepiece] and a filter block (Ploemopak, Leitz); a 100 W mercury lamp provided the illumination; appropriate filters for FITC, for rhodamine 6G and a heat filter (LeitzKG1) were used. The images of the pial microvascular network were televised with a DAGE MTI 1000 low-light level camera and recorded by a computer-based frame grabber (Pinnacle DC 10 plus, Avid Technology, MA, United States). A computer-assisted imaging software system was used to make off-line microvascular measurements (Lapi et al., 2020).

### Vascular permeability assays

In all animals, we evaluated four parameters, indices of microvascular damage:

A) Permeability increase, calculated as normalized gray levels (NGL): NGL = (I - Ir)/Ir, where Ir is the average baseline gray level at the end of the vessel filled with fluorescence (average of five windows located outside the blood vessels with the same windows being used throughout the experimental procedure), and I is the same parameter at the end of hypoperfusion or at the end of reperfusion. An MIP image program (MIP Image, CNR, Institute of Clinical Physiology, Pisa, Italy) was used to determine the gray levels ranging from 0 to 255 in five regions of interest (ROI) measuring 50 × 50 mm (×10 objective). To identify the same location of ROI during recordings along the microvascular networks, we used a computer-assisted device for XY movement of the microscope table.
B) ROS production was evaluated *in vivo* using DCFH-DA (Sigma-Aldrich, Milan, Italy) that permits the detection of oxidative species (Watanabe, 1998). This probe was dissolved in artificial cerebrospinal fluid (aCSF) composed (in mM) of 119.0 NaCl, 2.5 KCl, 1.3 MgSO_4_⋅ 7H_2_O, 1.0 NaH_2_PO_4_, 26.2 NaHCO_3_, 2.5 CaCl_2_, and 1.0 of glucose. A gaseous mixture containing 10% O_2_, 6% CO_2_, and 84% N_2_ was bubbled into the aCSF; pH 7.38 ± .02. The aCSF temperature was kept constant at 37.0°C ± .5°C.

The probe was superfused for 15 min during hypoperfusion or reperfusion; permeable to cells, it was hydrolyzed intracellularly to the DCFH carboxylate, trapped in the cells. Two electron oxidations of DCFH resulted in the formation of a fluorescent product, dichlorofluorescein (DCF), monitored with fluorescence microscopy. The fluorescence intensity of DCF was correlated directly to the intracellular ROS level, evaluated by an appropriate filter (522 nm) and quantified in NGL.

C) Leukocyte adhering to venular walls, evaluated as the number of cells on vessel walls that did not move over a 30-s observation period, was quantified in terms of number/100 mm of venular length (v.l.)/30, using × 20 and × 32, microscope objectives. The number of adherent leukocytes was assessed at two different times: before hypoperfusion (under baseline conditions) and at the end of reperfusion. The quantification was carried out by MIP image program.
D) Perfused capillaries, quantified as the length of capillaries showing blood flow (PCL), was assessed by MIP image in an area of 150–150 μm.

All the animals belonging to each experimental group, at the end of the respective treatments, were subjected to brain hypoperfusion and reperfusion. These data were compared with those evaluated in the age-matched adult SHRs, subjected to BCCAO and reperfusion, having taken into the account the time-interval of the specific treatment in the experimental groups, and in young SHRs, subjected to BCCAO and reperfusion, but still normotensive: in these two groups, we evaluated the microvascular parameters 5 min after the intravenous administration of the fluorescent tracer as baseline conditions.

### Statistical analysis

Data are presented as mean ± SEM. Due to the normality of the data (Kolmogorov–Smirnov method), the difference between groups was verified by one-way ANOVA, followed by the Bonferroni multiple comparisons test. Differences with *p* < .05 were considered statistically significant.

## Results

### Permeability increase

**FIGURE 2:**
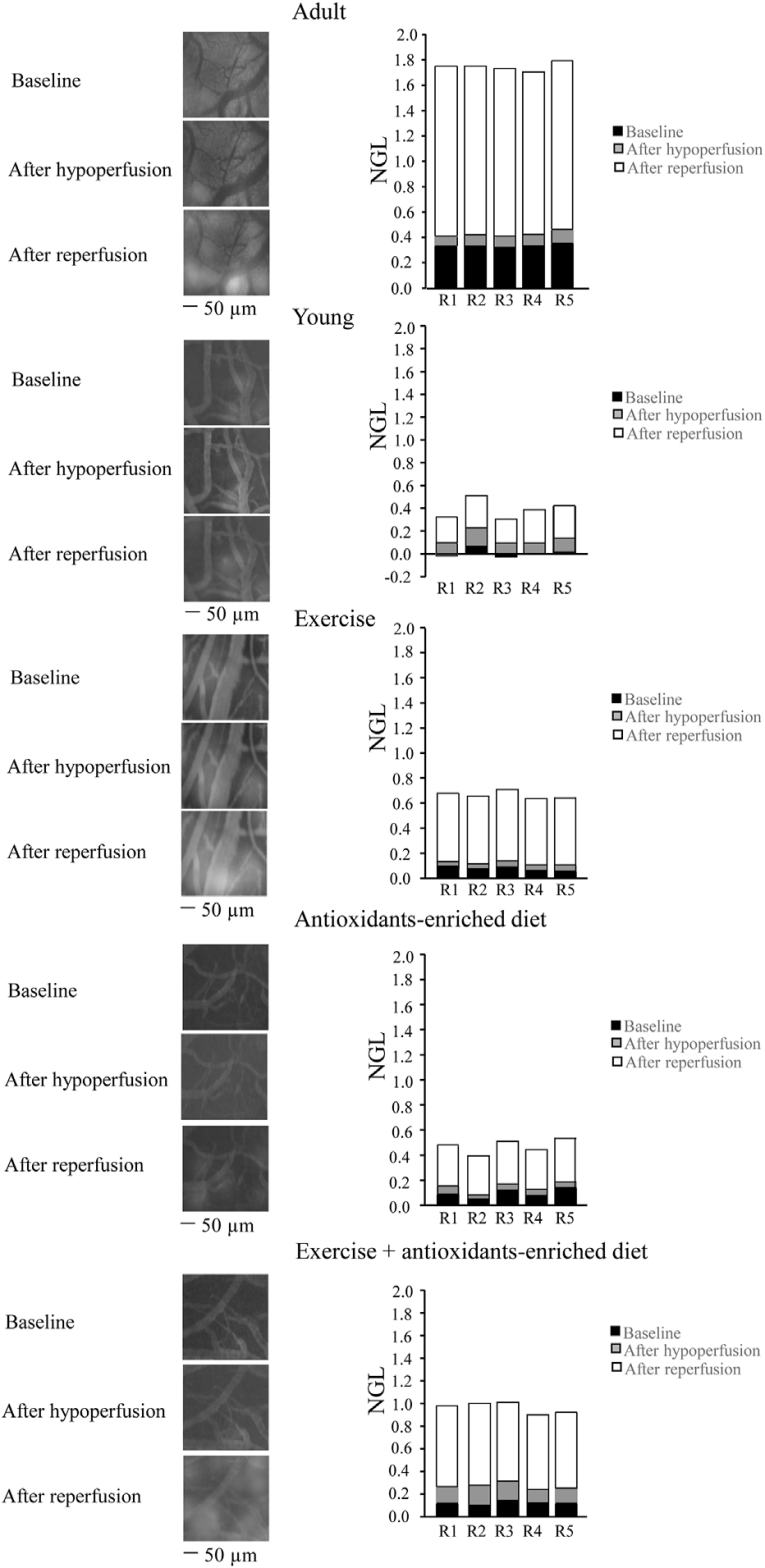
Changes in the increase of microvascular permeability evaluated *in vivo* in baseline conditions (baseline), after hypoperfusion and at the end of reperfusion in the different experimental groups. Right reports the graphs of the variations of the microvascular permeability quantified by the normalized gray levels (NGLs) for each animal (R1–R5), while left reports the corresponding video images obtained using a fluorescence microscope in an SHR belonging to each experimental group. *n* = 5 for each group.

Under baseline conditions, age-matched adult SHRs (adult group, A) showed a significant increase in fluorescent leakage compared with young SHRs (young group, Y) (NGL = .10 ± .01 and .04 ± .02, respectively, *p* < .01 *vs.* Y). At the end of hypoperfusion in adult SHRs, microvascular leakage was .11 ± .02 NGL (p = NS *vs.* baseline), while reperfusion caused a marked increase in microvascular permeability (NGL = 1.23 ± .03, *p* < .01 *vs.* baseline) (Figure 2).

Young SHRs showed a slight microvascular leakage (NGL = .04 ± .02, *p* < .01 *vs.* A group) under baseline conditions and at the end of BCCAO, presented an increase in leakage (NGL: .10 ± .02 (*p* < .01 *vs.* baseline), more pronounced at the end of the reperfusion (NGL: .26 ± .03, *p* < .01 *vs.* baseline and A group) (Figure 2). Exercising SHRs (E group) showed slight fluorescent leakage (NGL: .05 ± .01, *p* < .01 *vs.* A group) during baseline conditions. At the end of hypoperfusion, the fluorescent leakage was .08 ± .02 NGL (*p* < .01 *vs.* baseline), but the microvascular permeability increased at the end of reperfusion (NGL: .54 ± .02, *p* < .01 *vs.* baseline, A and Y groups) (Figure 2).

Antioxidants-enriched diet-treated SHRs (AED group) were protected from fluorescent leakage under baseline conditions, as observed in young SHRs (NGL: .04 ± .01, *p* < .01 *vs.* A group). At the end of BCCAO, NGL were .05 ± .01 (*p* < .01 *vs.* A, Y, and E groups) while after reperfusion, there was an increase: (NGL: .28 ± .0), lower compared with the other experimental groups (*p* < .01 *vs.* baseline, A and E groups) (Figure 2).

Under baseline conditions, physical exercise plus antioxidants-enriched diet-treated SHRs (E + AED group) showed a slight increase in leakage (NGL: .06 ± .02, *p* < .01 *vs.* A group). At the end of BCCAO, the microvascular permeability increased (NGL = .15 ± .02, *p* < .01 *vs.* baseline s, Y, E, and AED groups). After reperfusion, leakage was more pronounced (NGL = .69 ± .02, *p* < .01 *vs.* baseline, A, Y, E, and AED groups) (Figure 2).

#### Reactive oxygen species formation

**FIGURE 3:**
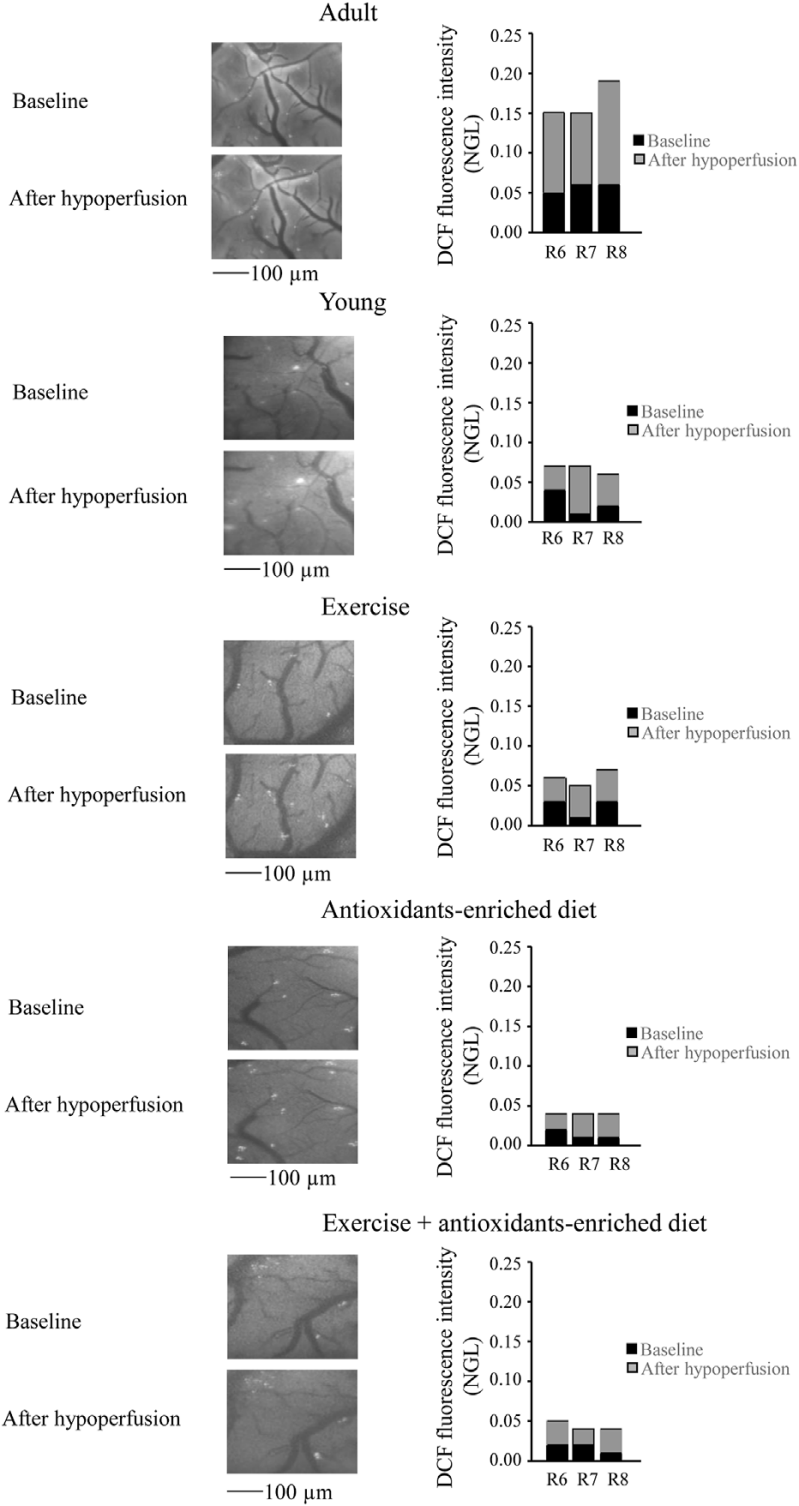
Reactive oxygen species (ROS) formation was evaluated *in vivo* by DCF fluorescence intensity measured by normalized gray levels (NGLs) in baseline conditions (*n* = 3), after 30 min of hypoperfusion (*n* = 3). Right reports the graphs showing the variations in each animal of each experimental group (R6–R8). Left reports the corresponding video images of the pial microcirculation obtained with a fluorescence microscope in a SHR rat belonging to each experimental group.

**FIGURE 4:**
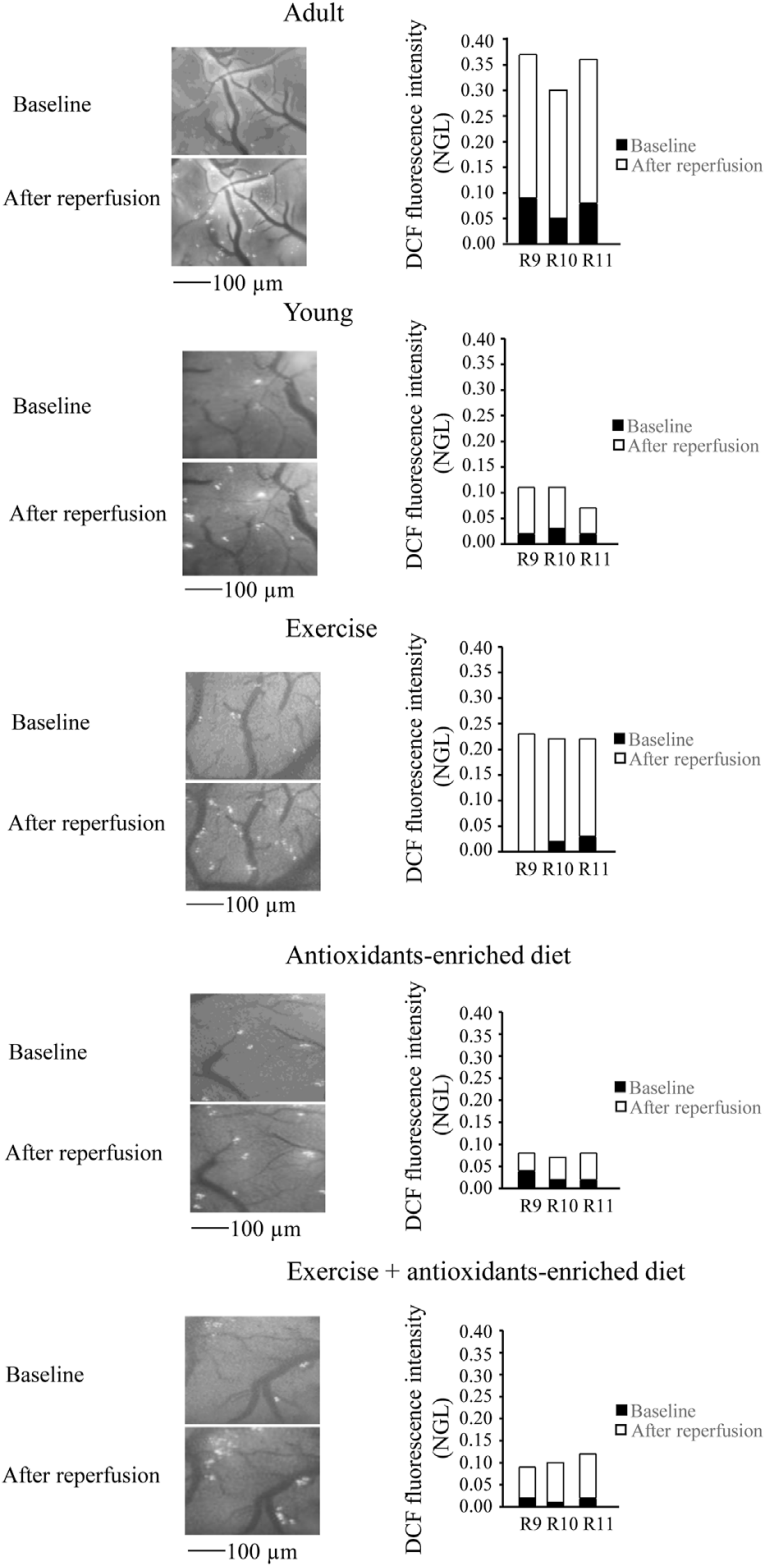
Reactive oxygen species (ROS) formation was evaluated *in vivo* by DCF fluorescence intensity measured by normalized gray levels (NGL) in baseline conditions (*n* = 3) and after 60 min of reperfusion (*n* = 3) in the different experimental groups. Right reports the graphs showing the variations in each animal of each experimental group (R9–R11). Left reports the corresponding video images of the pial microcirculation obtained with a fluorescence microscope in a SHR rat belonging to each experimental group.

Under baseline conditions, age-matched adult SHRs showed higher ROS formation than young SHRs (NGL = .10 ± .02 and .03 ± .02 NGL, respectively; *p* < .01 *vs.* Y group). At the end of hypoperfusion, DCF fluorescence intensity was .14 ± .02 NGL (*p* < .01 *vs.* Y group) (Figure 3), while at the end of reperfusion, there was a further increase (.30 ± .03 NGL, *p* < .01 *vs.* Y group) (Figure 4).

In young SHRs, under baseline conditions, the DCF fluorescence intensity was .03 ± .02 NGL (*p* < .01 *vs.* A group). After BCCAO, there was a slight increase in ROS formation (.09 ± .02 NGL, *p* < .01 *vs.* A group) (Figure 3); at the end of reperfusion, DCF fluorescence was .14 ± .02 NGL (*p* < .01 *vs.* baseline and A group) (Figure 4).

In the exercising SHRs group, under baseline condition, the ROS formation was minimal (NGL = .02 ± .01, *p* < .01 *vs.* A group). At the end of hypoperfusion, the DCF fluorescence intensity increased slightly (NGL = .05 ± .01, *p* < .01 *vs.* A and Y groups) (Figure 3), and after reperfusion, it was .22 ± .02 NGL (*p* < .01 *vs.* baseline, A and Y groups) (Figure 4).

The antioxidants-enriched diet treatment prevented the ROS formation: under baseline conditions, the DCF fluorescence intensity was minimal (NGL = .01 ± .01, *p* < .01 *vs.* A group). At the end of BCCAO (Figure 3) and reperfusion (Figure 4), ROS formation was .03 ± .01 NGL (*p* < .01 *vs.* A and Y groups) and .06 ± .01 NGL (*p* < .01 *vs.* baseline, A, Y and E groups) (Figure 3), respectively.

In the E + AED SHRs group, under baseline condition, the ROS formation was .02 ± .01 NGL (*p* < .01 *vs.* A group). After hypoperfusion (Figure 3) and reperfusion (Figure 4), the DCF fluorescence intensity was .04 ± .02 NGL (p = NS *vs.* baseline, *p* < .01 *vs.* A and Y groups) and .09 ± .01 NGL (*p* < .01 *vs.* A, Y and E groups), respectively.

### Leukocyte adhesion to the venular walls

**FIGURE 5:**
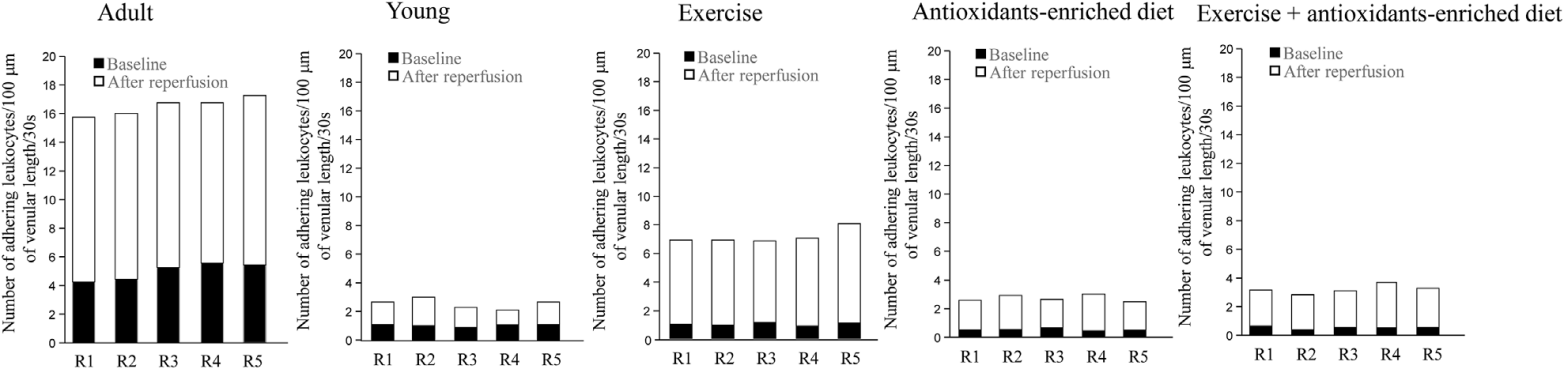
Leukocyte adhesion quantified by the number of leukocytes adhering to venular walls in baseline conditions and at the end of reperfusion is shown for each animal in different experimental groups. *n* = 5 for each group.

Under baseline conditions, in age-matched adult SHRs, there was significant leukocyte adhesion (5 ± 1/100 µm v.l./30 s) compared with leukocyte adhesion in young SHRs (1.0 ± .5, *p* < .01 *vs.* Y group) (Figure 5). At the end of reperfusion, the adhesion of leukocytes was pronounced in adult SHRs (11.5 ± 1.0/100 µm v.l./30 s), while in young SHRs, the number of adhered leukocytes was 1.5 ± 1.0/100 µm v.l./30 s, *p* < .01 *vs.* A group) (Figure 5).

Under baseline conditions, in exercising SHRs, the number of adhering leukocytes was 1.0 ± .5/100 µm v.l./30 s, *p* < .01 *vs.* A group). At the end of reperfusion, the adhering leukocytes were 6.0 ± 1.5/100 µm v.l./30 s, *p* < .01 *vs.* baseline, A and Y groups) (Figure 5).

In AED and E + AED SHRs groups, after reperfusion, the number of leukocytes adhering to the venular walls was 2.0 ± 1.0 and 2.5 ± 1.5/100 µm v.l./30 s, respectively; *p* < .01 *vs.* A and E groups) (Figure 5).

### Perfused capillary length

**FIGURE 6:**
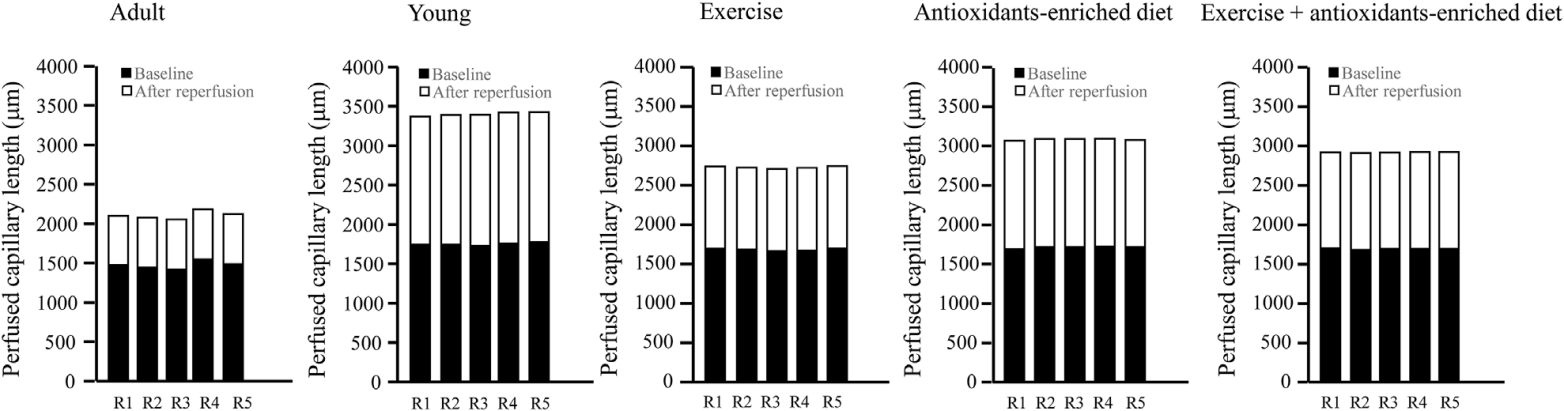
Supply of tissue blood flow was calculated as the length of the perfused capillaries in baseline conditions and after 60 min of reperfusion in the different experimental groups. The graphs showed the measure obtained in each animal in different experimental groups. *n* = 5 for each group.

It is interesting to note that under baseline conditions in all groups of animals, the capillaries were all perfused. However, age-matched adult SHRs showed a shorter average perfused capillary length (PCL) than young SHRs (1752 ± 88 μm, *p* < .01 *vs.* A group: 1,475 ± 70 μm) (Figure 6). At the end of reperfusion, in adult SHRs, there was a pronounced decrease in capillary perfusion by 57.0 ± 3.0% of baseline, while in young SHRs, the PCL was reduced by 5.8 ± 1.3% of baseline (*p* < .01 *vs.* A group) (Figure 6).

Under baseline conditions, the rats subjected to treatments showed a significant increase of PCL compared with untreated age-matched adult rats (by 14.2 ± 2.2%, 16.0 ± 1.8%, and 15 ± 3% in E, AED, and E + AED groups, respectively (*p* < .01 vs A group) (Figure 6).

At the end of reperfusion, capillary perfusion in exercising SHRs was significantly reduced by 38.0 ± 2.5% of baseline (*p* < .01 *vs.* A and Y groups) (Figure 6).

The treatment with the antioxidants-enriched diet partially preserved the perfused capillary length at the end of reperfusion because the reduction was by 20.0 ± 3.6% of baseline (*p* < .01 *vs.* A, Y, and E groups), while in the E + AED group, there was a decrease in PCL by 28 ± 2% of baseline after reperfusion (*p* < .01 *vs.* A and E groups) (Figure 6).

## Discussion

In the present study, we compared the effects of a physical exercise program with those induced by an antioxidant dietary supplement administered alone or in association on cerebral hypoperfusion and reperfusion injury in an experimental model of arterial hypertension, such as spontaneously hypertensive rats.

Previously, we showed that the same antioxidants-enriched diet produces hypotensive effects on the arterial blood pressure only during the administration period in a manner similar to that of physical activity (Del Seppia et al., 2022). However, in the SHRs only subjected to training, the hypotensive effect declined over time probably because of the oxidative stress induced by the physical exercise applied, which was progressively more intense, that mitigated the benefits on the cardiovascular system (Simioni et al., 2018). During the simultaneous administration of antioxidants, the side effects of physical activity can be mitigated so that the hypotensive effect of physical activity could be prolonged, as is the case in our physical exercise plus antioxidants-enriched diet group (Del Seppia et al., 2022).

The present data collected in the same animals used in Del Seppia et al. (2022) confirmed that in adult SHRs, arterial hypertension is accompanied by marked microvascular changes as previously described by Bobik (2005). In fact, under baseline conditions, prior to cerebral hypoperfusion and reperfusion injury, the pial microcirculation of adult SHRs presented several alterations, such as more conspicuous fluorescent dextran leakage, indicating increased permeability and increased formation of ROS, accompanied by a decrease of the perfused capillary length, when compared with the young SHRs. On average, the capillary network length was shorter in adult SHRs than in young SHRs, indicating a rarefication of microvascular networks in long-lasting arterial hypertension. However, at the end of reperfusion, there was an increase in microvascular permeability, in ROS formation, and in leukocytes adhering to the venular walls, accompanied by a significant reduction in perfused capillaries, and the changes were more marked in adult SHRs than in young SHRs.

It is known that regular physical exercise is a beneficial factor in the treatment of increased arterial blood pressure in human hypertension (Prasertsri et al., 2022), and human and experimental arterial hypertension are related to oxidative stress. Moreover, in a previous study, it was observed that physical exercise ameliorates the oxidative stress in SHRs treated with a high fat- or fructose-enriched diet (Szabó et al., 2021).

We subjected SHRs to a physical exercise program and antioxidants-enriched diet alone or in combination in order to assess whether in the pial microcirculation of SHRs, the two treatments in combination were able to reduce damage induced by hypoperfusion–reperfusion injury more than a single treatment.

Our results indicate that physical exercise protected the pial microcirculation under basal conditions, preventing the alterations observed in adult SHRs. This treatment partially protected from the damage due to brain hypoperfusion and reperfusion. At the end of reperfusion, the increase in microvascular permeability, the formation of free radicals and leukocytes adhesion were significantly reduced compared to those observed in age-matched adult SHRs, while 62% of the capillaries, on average, were perfused. The administration of natural antioxidant phytocomplex extracted from the apple variety Malus pumila Miller cv. Annurca, rich in catechins, was effective in reducing the pial microvascular impairment due to hypoperfusion and reperfusion injury. It is interesting to note that the antioxidants-enriched diet decidedly protected the pial microcirculation: the fluorescent leakage was significantly reduced compared to adult and young SHRs and exercising SHRs. The formation of free radicals and leukocyte adhesion were fully prevented. The capillaries were perfused to a larger extent (80% on average). These results support previous data, indicating that in hypertension, one of the main factors involved is the marked formation of ROS, effective in inducing microvascular damage.

We also administered a combination of progressively more intense physical exercise and antioxidants’ supplement. Our results indicate that all three treatments were effective in preventing brain damage due to cerebral hypoperfusion and reperfusion. However, the microvascular parameters improved to different extents, according to the different treatments. It was surprising that combining physical exercise and antioxidants-enriched diet, the pial microcirculation was less preserved by leakage fluorescence than after the single treatment in both hypoperfusion and reperfusion conditions, and the ROS formation was reduced with respect to physical exercise alone, but increased in comparison with an antioxidants-enriched diet alone. A similar effect was detected in leukocyte adhesion. Moreover, the combined treatments were effective in preserving the capillary perfusion to a higher extent compared to physical exercise alone, but was less effective compared to antioxidants-enriched diet alone.

To explain these unexpected results, we can observe that while moderate physical exercise reduces oxidative stress, acute exercise increases oxidative stress with negative physiological consequences (Pingitore et al., 2015). Our physical exercise program consisted of gradually increasing levels of physical activity, probably producing an enhancement of oxidative stress mainly in the last week, which cannot be compensated by the administration of concomitant antioxidants. Moreover, it is known that human subjects performing physical activity under exogenous antioxidant treatment show an impairment of different physiological parameters (for review, see Brisswalter and Louis, 2014; Peternelj and Coombes, 2011). However, in our experiments, physical exercise and an antioxidants-enriched diet in association determine protective effects during the development of a hypertensive status in a condition of hypoperfusion and reperfusion, even if it seems necessary to increase the dosage of antioxidants during an increasing physical activity to obtain better vascular protection.

### Permission to reuse and copyright

Figures, tables, and images will be published under a Creative Commons CC-BY license, and permission must be obtained for use of copyrighted material from other sources (including republished/adapted/modified/partial figures and images from the Internet). It is the responsibility of the authors to acquire the licenses, to follow any citation instructions requested by third-party rights holders, and cover any supplementary charges.

## Data Availability

The original contributions presented in the study are included in the article/Supplementary Material. Further inquiries can be directed to the corresponding author.
